# Temporal trend of serum vitamin profiles and the association with non-alcoholic fatty liver disease

**DOI:** 10.3389/fnut.2026.1712392

**Published:** 2026-05-08

**Authors:** Chunyu Hu, Junliang Zhou, Li Sun, Zongtao Chen

**Affiliations:** Department of Health Management, The First Affiliated Hospital of Army Medical University (Southwest Hospital), Chongqing, China

**Keywords:** joint effect, non-alcoholic fatty liver disease, restricted cubic spline, serum vitamin, temporal trend

## Abstract

**Background:**

Few studies have simultaneously examined temporal trends in multiple serum vitamins and their associations with non-alcoholic fatty liver disease (NAFLD), especially in non-Western populations. This study aimed to characterize temporal trends of serum vitamins (A, B9, B12, D, E) among Chinese adults undergoing health examinations (2015–2023) and explore their associations with NAFLD.

**Methods:**

Subjects were extracted from the electronic health records of the Department of Health Management, the First Affiliated Hospital of Army Medical University. Vitamin levels were quantified via electrochemical methods. NAFLD was diagnosed using abdominal ultrasound. Mean vitamin levels and 95% confidence intervals (CIs) were calculated and compared across 9 years overall and in subgroups (age, sex, BMI, and NAFLD status). Odds ratios (ORs) and 95% CIs for the associations between vitamin quintile groups and NAFLD risk were assessed by logistic regression. Dose-response relationships were explored using the restricted cubic spline (RCS) approach. Joint effects of vitamins B9 and B12 were also evaluated.

**Results:**

75,401 adults with complete data on vitamins were included. Serum vitamins A, B9, B12, 25(OH)D, and E changed annually by −0.007, −0.223, −4.043, −0.577, and −0.075 (all *P*_trend_ < 0.05). Stratified analyses revealed the lowest serum vitamin levels among women, individuals aged ≥60 years, and those with overweight/obesity or NAFLD. 67,356 (27.515% NAFLD) eligible participants were used for association analysis. Lower vitamin levels were associated with an increased NAFLD risk. The RCS curves indicated non-linear relationships between each vitamin and NAFLD status (All *P*_non − linear_ < 0.001). Higher vitamins B9 and B12 levels may correlate with lower NAFLD prevalence.

**Conclusion:**

Our findings indicated a downward trend of serum vitamins A, B9, B12, D, and E during the 9-year period among Chinese health examination adults, especially among senior women.

## 1 Introduction

Vitamins are essential micronutrients that underpin diverse physiological functions, with deficiencies or imbalances linked to metabolic and chronic diseases (1). Vitamin A, a fat-soluble vitamin, regulates vision, cell differentiation, immune function, and glucose-lipid metabolism; its dysregulation may disrupt hepatic lipid homeostasis (2). Vitamin B9 (folate) mediates one-carbon metabolism, supporting nucleic acid synthesis, amino acid balance, and immune responses (3), while also influencing hepatic oxidative stress, inflammation, and lipid metabolism (4–6). Vitamin B12 (cobalamin), another one-carbon metabolism cofactor, facilitates folate transport and lipid metabolism (3, 7, 8). Low levels of vitamin B12 elevate homocysteine (a CVD risk factor), impair mitochondrial function, and are associated with megaloblastic anemia, liver diseases like hepatocellular carcinoma, and increased all-cause mortality, often co-occurring with B9 deficiency (9–14). Vitamin D, obtained via skin synthesis or diet, modulates calcium-phosphate balance, immunity, and metabolism, with low serum 25-hydroxyvitamin D [25(OH)D] linked to metabolic syndrome and CVD risk (15–20). Vitamin E, a natural antioxidant, effectively inhibits lipid oxidation and shows therapeutic potential for liver health (21). Given their multifaceted roles, understanding serum vitamin profiles and their dynamics is integral to assessing public health and disease risk.

Non-alcoholic fatty liver disease (NAFLD), now also termed metabolic dysfunction-associated steatotic liver disease (MASLD), is a hepatic disorder tied to cardiometabolic dysregulation (22). Given that patients meeting the criteria for NAFLD or MASLD are highly consistent (23–25), NAFLD is used here for historical and clinical familiarity. Globally, NAFLD affects over 30% of the population, associated with hepatocellular carcinoma and increased all-cause mortality (26–29). Rising obesity rates have driven its incidence, creating a substantial global health burden (30). Existing research links specific vitamins to NAFLD (31). Low vitamin B9 and B12 correlate with histological severity in NAFLD patients (13), 25(OH)D may link to NAFLD risk with ethnic difference (32, 33), and vitamin E supplementation shows promise for reducing hepatic inflammation (21).

Vitamin temporal changes differ by population and are shaped by age, diet, socioeconomic status, gender, and geography. Global under-20 vitamin A deficiency (VAD)-related mortality fell sharply from 1990 to 2021 due to childhood supplementation, yet regional disparities remained. High-sociodemographic index (SDI) countries like the US and Sweden reached near-zero age-standardized VAD rates by 2021, while low-SDI nations such as Somalia had the highest age-standardized disability-adjusted life year rates, highlighting unmet needs in resource-limited regions (34). US adults showed improved long-term vitamin B9 status 2003–2016, likely due to grain fortification, though serum vitamin B9 insufficiency followed a U-shaped trend (35). Their serum vitamin B12 levels also rose during 2003–2016 (35). For vitamin D, US overall levels increased from 2001 to 2018, but young adults (20–39 years) and low-income groups had worsening deficiency, while adults ≥60 years saw improvement, and northern Sweden had no clear trend with fluctuating 25(OH)D during 1986–2014 (36, 37). Vitamin E in US adults had a modest serum level decline from 2003 to 2016 but negligible deficiency rates (35). Notably, micronutrient laboratory testing, including vitamins, rose substantially in Switzerland 2012–2018, reflecting growing clinical and public interest in vitamin-related health impacts (38). Understanding population-level vitamin trends is critical for assessing nutritional status and guiding public health actions.

Despite growing research, critical gaps persist. Few studies have examined temporal trends in multiple serum vitamins alongside their collective associations with NAFLD risk in general populations, particularly in non-Western populations. Therefore, this study aims to: (1) characterize temporal trends in serum levels of vitamins A, B9, B12, D, and E among Chinese adults undergoing health examinations from 2015 to 2023; and (2) investigate their associations with NAFLD risk. By integrating longitudinal trends with disease risk, this research seeks to inform public health strategies and identify nutritional targets for NAFLD prevention and management.

## 2 Methods

### 2.1 Study participants

Health examination data, including socio-demographics, anthropometric characteristics, and laboratory tests, were retrieved from the electronic health records database of the Department of Health Management, the First Affiliated Hospital of Army Medical University. The study was approved by the Ethics Committee of the same hospital in accordance with the Helsinki Declaration (No. [A]KY202227). All subjects provided written informed consent to participate.

A total of 756,859 Chinese individuals who underwent health check-ups with completed abdominal ultrasonography between 2015 and 2023 were initially included. Participants were excluded if: (1) aged < 18 years (*n* = 3079); (2) with missing sex information (*n* = 14); or (3) without serum vitamin testing (*n* = 678,365). Ultimately, 75,401 participants were included in the temporal trend analysis. For subsequent association analysis, only the first record was retained for participants with multiple entries (*n* = 67,356).

### 2.2 Measurement of serum vitamins

Serum samples were collected, stored, and processed following standardized protocols. Venous blood was drawn in the morning after a 10-h fast and centrifuged within 2 h of collection. Electrochemical stripping voltammetry was employed for the simultaneous quantitative determination of multiple vitamin components, including vitamins A, B_9_, B_12_, D, and E, using the LK3000V Vitamin Detector (Tianjin Lanbiao Electronic Technology Development Co., Ltd.). The measured vitamin D biomarker was 25(OH)D. The instrument achieved a minimum detectable concentration of 1.7 × 10^−12^ mol/L. Qualitative identification was performed based on the peak potential of the stripping polarization curve, while quantitative analysis relied on the linear relationship between peak current and analyte concentration. To address the reliability and accuracy of vitamin detection, the laboratory regularly participated in external quality assessment (EQA) schemes organized by the Chongqing Center for Clinical Laboratory, with at least one participation per year to ensure the validity and stability of the detection system. Outliers were defined as values exceeding three standard deviations (SD) from the overall mean and were replaced with the corresponding mean ± 3 × SD, as appropriate.

For analyzing associations with NAFLD, vitamin levels were stratified into quintile groups. For vitamin A, the quintile groups were Q1 [0.45, 0.70], Q2 (0.70, 0.77], Q3 (0.77, 0.87], Q4 (0.87, 0.97], and Q5 (0.97, 1.40] μmol/L; for vitamin B9, Q1 [8.60, 15.40], Q2 (15.40, 18.39], Q3 (18.39, 19.34], Q4 (19.34, 21.06], and Q5 (21.06, 28.26] nmol/L; for vitamin B12, Q1 [174.09, 321.58], Q2 (321.58, 379.85], Q3 (379.85, 413.18], Q4 (413.18, 465.23], and Q5 (465.23, 613.97] pg/mL; for 25(OH)D, Q1 [14.03, 31.22], Q2 (31.22, 38.11], Q3 (38.11, 41.96], Q4 (41.96, 47.32], and Q5 (47.32, 65.18] nmol/L; and for vitamin E, Q1 [8.77, 11.25], Q2 (11.25, 12.66], Q3 (12.66, 13.47], Q4 (13.47, 14.36 ], and Q5 (14.36, 18.13] μg/mL.

### 2.3 Ascertainment of NAFLD

Liver biopsy, the gold standard for NAFLD diagnosis, is restricted by its invasiveness and high cost. Abdominal ultrasound is more accessible and less expensive than computed tomography (CT) or magnetic resonance imaging (MRI); thus, NAFLD status was assessed via abdominal ultrasound examination by experienced radiologists. NAFLD was defined according to the hepatorenal echo contrast, brightness, deep attenuation, and vascular sharpness, in accordance with Chinese guidelines for NAFLD diagnosis (39).

### 2.4 Definition of other variables

Individual information, including age, sex, race, marital status, height, weight, waist circumference, blood pressure, cholesterol levels, fasting blood glucose, aspartate aminotransferase (AST), alanine aminotransferase (ALT), alkaline phosphatase (ALP), and gamma-glutamyl transpeptidase (GGT), was obtained. Age was classified as 18–44, 45–60, and ≥60 years. Ethnicity included Han Chinese and ethnic minorities. Marital status was categorized into married/cohabiting and unmarried/divorced. Body mass index (BMI) was calculated as weight in kilograms divided by height in meters squared (kg/m^2^) and then categorized as < 24, 24–28, and ≥28 kg/m^2^. Hypertension was defined as systolic blood pressure/diastolic blood pressure (SBP/DBP) ≥140/90 mmHg. Diabetes was defined as fasting blood glucose ≥126 mg/dL. Dyslipidemia was defined as total cholesterol (TC) ≥240 mg/dL, and/or triglycerides (TG) ≥200 mg/dL, and/or low-density lipoprotein cholesterol (LDL-C) ≥160 mg/dL, and/or high-density lipoprotein cholesterol (HDL-C) < 40 mg/dL.

### 2.5 Statistical analysis

Participants were categorized by calendar year to compare basic characteristics. Chi-squared test was used for categorical variables, and the ANOVA or *t*-test was used for continuous variables. The estimates of NAFLD prevalence were age-standardized to the Seventh China Population Census in 2020, using the age groups 18–44, 45–64 years, and ≥65 years. Mean vitamin levels with 95% confidence intervals (CIs) were calculated and compared across the 9-year period, both overall and in subgroups (age, sex, BMI, and NAFLD status), with trends assessed via partial Mann-Kendall tests. For further validation, Joinpoint statistical software (40) was used to identify temporal trends in vitamin levels. Under a linear model, vitamin levels changed at a constant value per year. Joinpoint regressions with heteroscedastic and correlated errors were used to determine trends in vitamin levels, allowing 1 joinpoint. The joinpoint location was identified by grid search where applicable. The best-fitting model was selected by conducting 4,499 permutation tests based on a Monte Carlo method. The parametric method was used for stratified trend analyses by age, sex, BMI, and NAFLD.

Logistic regression models were applied to estimate odds ratios (ORs) and 95% CIs for the associations between serum vitamin quintile groups and NAFLD risk, with participants in the lowest quintile intervals as the reference group. In model 1, we adjusted for the year of health examination, age, and sex. In model 2, race and marital status were added. In model 3, BMI group, hypertension, diabetes, and dyslipidemia were further adjusted. Multicollinearity was assessed using the variance inflation factor (VIF). To further investigate the dose-response relationship, restricted cubic spline (RCS) analyses were performed with adjustment for the same covariates as Model 3. Four knots were placed at the 5th, 35th, 65th, and 95th percentiles, with the reference value set at the 10th percentile. Additionally, we conducted a joint analysis based on the combined status of vitamin B9 and B12. Each vitamin level was classified as high or low using the median value, forming four groups (low B9 and low B12, low B9 and high B12, high B9 and low B12, high B9 and high B12), with the low B9 and low B12 group as the reference.

All tests were two-sided, with *P* < 0.05 considered statistically significant. All analyses were performed using *R* version 4.3.1 (https://www.r-project.org/).

## 3 Results

### 3.1 Basic characteristics

Among 75,401 adults with complete serum vitamin profile data (Table 1), the mean (SD) age was 47.335 (12.219) years, with 37,350 (49.535%) being men and 97.806% being Chinese Han. From 2015 to 2023, the estimated age-standardized prevalence of NAFLD fluctuated without a significant downward trend (*P*_trend_ = 0.466). Among 67,356 eligible adults with complete data on serum vitamin profiles and NAFLD (Supplementary Table S1), the mean (SD) age was 47.066 (12.391) years, with 32,795 (48.689%) being men. Compared with the non-NAFLD group, participants with NAFLD were older and had a higher prevalence of overweight/obesity, hypertension, diabetes, and dyslipidemia.

**Table 1:** Basic characteristics in the health examination population, 2015–2023.

Characteristics	2015	2016	2017	2018	2019	2020	2021	2022	2023	*P* value
	(*n =* 9,805)	(*n =* 6,535)	(*n =* 6,997)	(*n =* 6,697)	(*n =* 10,205)	(*n =* 8,859)	(*n =* 10,622)	(*n =* 8,376)	(*n =* 7,305)	
**Crude prevalence, 1/100,000**	29,056.604	27,467.483	27,769.044	26,653.726	28,084.272	28,976.182	26,718.132	28,330.950	29,514.031	< 0.001
**Age-standardized rate^a^**	29,099.940 (28,030.777, 30,203.106)	27,649.812 (26,373.425, 28,977.388)	27,557.998 (26,337.428, 28,824.258)	26,076.687 (24,866.045, 27,333.238)	27,161.202 (26,146.128, 28,208.091)	27,724.252 (26,622.913, 28,862.659)	25,389.89 (24,414.888, 26,397.305)	26,996.075 (25,864.6, 28,169.417)	28,090.337 (26,868.6, 29,358.992)	0.152^b^
**Age, year**	45.313 (11.889)	44.952 (12.163)	45.584 (12.389)	46.906 (12.317)	47.623 (11.832)	47.789 (11.999)	48.486 (11.837)	49.346 (12.259)	49.318 (12.744)	< 0.001
Age group									
18-44	4,738 (48.322%)	3,217 (49.227%)	3,172 (45.334%)	2,692 (40.197%)	3,689 (36.149%)	3,131 (35.343%)	3,471 (32.677%)	2,632 (31.423%)	2,353 (32.211%)	< 0.001
45-60	3,836 (39.123%)	2,494 (38.164%)	2,869 (41.003%)	2,986 (44.587%)	4,998 (48.976%)	4,451 (50.243%)	5,630 (53.003%)	4,333 (51.731%)	3,569 (48.857%)	
≥60	1,231 (12.555%)	824 (12.609%)	956 (13.663%)	1,019 (15.216%)	1,518 (14.875%)	1,277 (14.415%)	1,521 (14.319%)	1,411 (16.846%)	1,383 (18.932%)	
**Men**, ***n*** **(%)**	5,101 (52.024%)	3,237 (49.533%)	3,559 (50.865%)	3,406 (50.859%)	5,108 (50.054%)	4,462 (50.367%)	5,151 (48.494%)	3,901 (46.574%)	3,425 (46.886%)	< 0.001
**Chinese Han**, ***n*** **(%)**	9,666 (98.582%)	6,446 (98.638%)	6,938 (99.157%)	6,631 (99.014%)	10,081 (98.785%)	8,746 (98.724%)	10,253 (96.526%)	8,039 (95.977%)	6,947 (95.099%)	< 0.001
**Married/cohabiting**, ***n*** **(%)**	5,547 (56.573%)	3,461 (52.961%)	3,791 (54.18%)	3,248 (48.499%)	4,701 (46.066%)	3,586 (40.479%)	2,741 (25.805%)	1,392 (16.619%)	1,098 (15.031%)	< 0.001
**Vitamin A (umol/L)**	0.867 (0.152)	0.869 (0.166)	0.865 (0.178)	0.827 (0.173)	0.83 (0.186)	0.836 (0.187)	0.83 (0.188)	0.817 (0.19)	0.814 (0.193)	< 0.001
**Vitamin B9 (nmol/L)**	19.361 (2.737)	19.079 (3.053)	18.873 (3.158)	18.399 (3.201)	18.259 (3.266)	18.17 (3.256)	18.01 (3.233)	17.686 (3.267)	17.592 (3.309)	< 0.001
**Vitamin B12 (pg/mL)**	407.239 (63.035)	410.66 (68.514)	406.315 (68.714)	381.794 (74.849)	389.307 (69.991)	391.843 (70.688)	388.638 (71.723)	382.021 (73.264)	380.216 (74.659)	< 0.001
**25(OH)D (nmol/L)**	42.766 (8.813)	41.653 (8.093)	40.853 (7.977)	37.614 (8.708)	38.305 (7.895)	39.088 (7.747)	38.729 (7.643)	38.016 (7.79)	37.892 (7.896)	< 0.001
**Vitamin E (ug/mL)**	12.969 (1.514)	13.195 (1.516)	13.04 (1.705)	13.067 (1.748)	12.826 (1.734)	12.742 (1.718)	12.719 (1.818)	12.603 (1.879)	12.584 (1.914)	< 0.001
**BMI (kg/m** ^2^ **)**	24.432 (3.53)	24.172 (3.467)	23.996 (3.454)	24.252 (3.49)	24.544 (3.512)	24.319 (3.417)	24.343 (3.437)	24.308 (3.454)	24.409 (3.524)	< 0.001
BMI group									
< 24	4,430 (47.253%)	3,215 (50.329%)	3,507 (51.415%)	3,186 (48.753%)	4,526 (45.634%)	4,116 (48.017%)	4,930 (48.428%)	3,884 (48.495%)	3,334 (47.486%)	< 0.001
24-28	3,603 (38.432%)	2,327 (36.428%)	2,489 (36.49%)	2,448 (37.46%)	3,933 (39.655%)	3,357 (39.162%)	3,882 (38.134%)	3,058 (38.182%)	2,682 (38.2%)	
≥28	1,342 (14.315%)	846 (13.244%)	825 (12.095%)	901 (13.787%)	1,459 (14.711%)	1,099 (12.821%)	1,368 (13.438%)	1,067 (13.323%)	1,005 (14.314%)	
**WC (cm)**	82.738 (10.307)	82.763 (10.177)	83.159 (10.665)	82.996 (10.576)	83.434 (10.345)	82.418 (10.289)	82.455 (9.951)	83.345 (10.168)	82.429 (10.458)	< 0.001
**SBP (mmHg)**	122.038 (18.569)	121.852 (18.151)	125.197 (18.059)	124.128 (18.463)	124.532 (18.517)	124.531 (18.299)	124.782 (17.945)	125.962 (17.7)	126.402 (18.49)	< 0.001
**DBP (mmHg)**	76.726 (12.618)	76.258 (12.333)	77.887 (12.555)	77.376 (12.744)	77.81 (12.521)	76.856 (12.083)	76.915 (12.035)	77.83 (11.95)	78.604 (12.255)	< 0.001
**Fasting glucose (mg/dL)**	101.994 (25.635)	100.573 (23.431)	100.14 (26.177)	102.405 (25.693)	104.195 (26.699)	103.198 (26.211)	105.622 (28.08)	106.632 (28.524)	103.463 (27.982)	< 0.001
**TC (mg/dL)**	196.563 (38.747)	195.267 (38.974)	194.215 (38.925)	197.715 (38.449)	199.192 (39.266)	199.479 (39.356)	196.452 (38.894)	195.936 (38.609)	198.635 (39.342)	< 0.001
**TG (mg/dL)**	153.958 (130.36)	154.651 (130.974)	154.977 (127.346)	155.078 (127.856)	155.663 (122.591)	152.689 (125.618)	145.475 (121.475)	143.394 (119.274)	146.994 (123.13)	< 0.001
**LDL-C (mg/dL)**	97.934 (22.766)	96.52 (22.569)	101.194 (23.506)	108.749 (27.339)	122.814 (27.971)	120.553 (28.299)	121.128 (27.861)	121.54 (27.919)	122.72 (27.287)	< 0.001
**HDL-C (mg/dL)**	52.998 (12.272)	53.161 (12.152)	53.648 (12.904)	52.896 (12.564)	49.78 (11.994)	52.171 (12.323)	50.799 (12.055)	52.919 (12.265)	54.777 (12.036)	< 0.001
**Hypertension**, ***n*** **(%)**	985 (19.899%)	1,175 (18.319%)	1,553 (22.612%)	1,508 (22.935%)	2,296 (23.052%)	1,819 (21.141%)	2,164 (21.122%)	1,842 (22.848%)	1,817 (25.824%)	< 0.001
**Diabetes**, ***n*** **(%)**	595 (6.13%)	379 (5.875%)	434 (6.311%)	461 (6.993%)	794 (7.883%)	650 (7.424%)	845 (8.046%)	731 (8.851%)	602 (8.358%)	< 0.001
**Dyslipidemia**, ***n*** **(%)**	3,197 (33.624%)	2,121 (33.523%)	2,294 (33.94%)	2,320 (35.561%)	4,088 (40.766%)	3,191 (36.523%)	3,866 (36.988%)	2,748 (33.427%)	2,318 (32.212%)	< 0.001
**GGT (IU/L)**	39.630 (52.289)	38.014 (49.052)	38.525 (46.801)	37.31 (47.694)	37.927 (49.442)	37.236 (48.182)	34.328 (41.015)	34.131 (43.777)	35.32 (42.716)	< 0.001
**AST (IU/L)**	26.816 (27.215)	27.091 (18.426)	26.634 (20.142)	25.937 (18.683)	25.301 (17.624)	25.577 (13.207)	25.113 (15.426)	25.461 (14.542)	24.408 (11.028)	< 0.001
**ALT (IU/L)**	28.27 (30.781)	28.39 (31.028)	27.683 (21.111)	27.17 (27.038)	26.517 (25.941)	26.311 (19.717)	25.375 (20.485)	25.671 (20.959)	25.164 (18.765)	< 0.001
**ALP (IU/L)**	89.571 (27.885)	87.792 (29.222)	88.891 (28.064)	84.669 (26.521)	87.743 (29.279)	83.773 (26.651)	77.522 (23.973)	78.352 (24.422)	77.611 (24.44)	< 0.001

^a^The estimates of NAFLD prevalence were age-standardized to the Seventh China Population Census in 2020, using the age groups 18-44, 45-64, and ≥65 years.

^b^Temporal trends in NAFLD prevalence across 2015–2023 were analyzed using the partial Mann-Kendall test. ANOVA (continuous variables) and Chi-square tests (categorical variables) were used to explore the differences in all basic indicators across years.

### 3.2 Temporal trends of serum vitamin profiles

The temporal trends of serum vitamin profiles were compared across 9 years (Table 2). Annual changes in serum vitamin concentrations, as determined by the partial Mann–Kendall test, showed monotonic trends throughout the 9-year study period. These changes indicated the long-term direction and magnitude of variations in vitamin levels. Annual mean values from 2015 to 2023, however, represented cross-sectional measures with modest year-to-year fluctuations. Mean vitamin A decreased from 0.867 (95% CI: 0.866, 0.868) to 0.814 (95% CI: 0.812, 0.815) μmol/L, with an annual change of −0.007 (95% CI: −0.010, −0.003; *P*_trend_ = 0.009) from 2015 to 2023. Using the mean vitamin A from 2015 as a reference, the changes ranged from 0.002 to −0.054 and differed significantly starting in 2018. Mean vitamin B9 decreased from 19.361 (95% CI: 19.342, 19.381) to 17.592 (95% CI: 17.569, 17.616) nmol/L, with an annual change of −0.223 (95% CI: −0.247, −0.167; *P*_trend_ < 0.001). Relative to the 2015 mean, changes ranged from −0.282 to −1.769. Mean vitamin B12 decreased from 407.239 (95% CI: 406.789, 407.689) to 380.216 (95% CI: 379.683, 380.749) pg/mL, with an annual change of −4.043 (95% CI: −4.818, −0.473; *P*_trend_ = 0.016). Relative to the 2015 mean, changes ranged from 3.421 to −27.023 and differed significantly starting in 2018. Mean 25(OH)D decreased from 42.766 (95% CI: 42.703, 42.829) to 37.892 (95% CI: 37.835, 37.948) nmol/L, with an annual change of −0.577 (95% CI: −0.802, −0.103; *P*_trend_ = 0.029). Relative to the 2015 mean, changes ranged from −1.113 to −4.874. Mean vitamin E decreased from 12.969 (95% CI: 12.958, 12.980) to 12.584 (95% CI: 12.570, 12.597) μg/mL, with an annual change of −0.075 (95% CI: −0.107, −0.045; *P*_trend_ = 0.005) from 2015 to 2023. Using the mean vitamin A from 2015 as a reference, the changes ranged from 0.225 to −0.386 and differed significantly starting in 2018. Results from joinpoint regression were generally consistent with the above findings, showing overall decreasing trends. Notably, they indicated that vitamins B9 and 25(OH)D declined rapidly during 2015–2018 but slowed thereafter from 2018 to 2023 (Supplementary Figures S1–S5).

**Table 2:** Magnitude of changes in mean vitamin levels across the calendar year, 2015–2023.

Years	Mean [SE] (95% CI)	Difference	*P* value for difference in vitamin^a^
Vitamin A (umol/L)			
2015	0.867 [0.001] (0.866, 0.868)	0 (Reference)	–
2016	0.869 [0.001] (0.868, 0.87)	0.002	0.999
2017	0.865 [0.001] (0.863, 0.866)	−0.003	0.993
2018	0.827 [0.001] (0.826, 0.828)	−0.040	< 0.001
2019	0.830 [0.001] (0.829, 0.832)	−0.037	< 0.001
2020	0.836 [0.001] (0.834, 0.837)	−0.031	< 0.001
2021	0.830 [0.001] (0.828, 0.831)	−0.038	< 0.001
2022	0.817 [0.001] (0.815, 0.818)	−0.050	< 0.001
2023	0.814 [0.001] (0.812, 0.815)	−0.054	< 0.001
Overall *P* value for trend ^b^	0.009		
Vitamin B9 (nmol/L)			
2015	19.361 [0.010] (19.342, 19.381)	0 (Reference)	–
2016	19.079 [0.011] (19.057, 19.101)	−0.282	< 0.001
2017	18.873 [0.012] (18.85, 18.895)	−0.488	< 0.001
2018	18.399 [0.012] (18.376, 18.422)	−0.962	< 0.001
2019	18.259 [0.012] (18.235, 18.282)	−1.103	< 0.001
2020	18.17 [0.012] (18.146, 18.193)	−1.192	< 0.001
2021	18.01 [0.012] (17.987, 18.034)	−1.351	< 0.001
2022	17.686 [0.012] (17.663, 17.71)	−1.675	< 0.001
2023	17.592 [0.012] (17.569, 17.616)	−1.769	< 0.001
Overall *P* value for trend ^b^	< 0.001		
Vitamin B12 (pg/mL)			
2015	407.239 [0.230] (406.789, 407.689)	0 (Reference)	–
2016	410.660 [0.250] (410.171, 411.149)	3.421	0.060
2017	406.315 [0.250] (405.824, 406.805)	−0.924	0.996
2018	381.794 [0.273] (381.260, 382.328)	−25.445	< 0.001
2019	389.307 [0.255] (388.808, 389.807)	−17.931	< 0.001
2020	391.843 [0.257] (391.338, 392.348)	−15.396	< 0.001
2021	388.638 [0.261] (388.126, 389.150)	−18.601	< 0.001
2022	382.021 [0.267] (381.498, 382.544)	−25.218	< 0.001
2023	380.216 [0.272] (379.683, 380.749)	−27.023	< 0.001
Overall *P* value for trend^b^	0.016		
25(OH)D (nmol/L)			
2015	42.766 [0.032] (42.703, 42.829)	0 (Reference)	–
2016	41.653 [0.029] (41.595, 41.711)	−1.113	< 0.001
2017	40.853 [0.029] (40.797, 40.910)	−1.912	< 0.001
2018	37.614 [0.032] (37.552, 37.676)	−5.152	< 0.001
2019	38.305 [0.029] (38.249, 38.361)	−4.461	< 0.001
2020	39.088 [0.028] (39.032, 39.143)	−3.678	< 0.001
2021	38.729 [0.028] (38.674, 38.784)	−4.037	< 0.001
2022	38.016 [0.028] (37.960, 38.072)	−4.750	< 0.001
2023	37.892 [0.029] (37.835, 37.948)	−4.874	< 0.001
Overall *P* value for trend^b^	0.029		
Vitamin E (ug/mL)			
2015	12.969 [0.006] (12.958, 12.980)	0 (Reference)	–
2016	13.195 [0.006] (13.184, 13.205)	0.225	0.000
2017	13.04 [0.006] (13.027, 13.052)	0.070	0.188
2018	13.067 [0.006] (13.054, 13.079)	0.097	0.012
2019	12.826 [0.006] (12.813, 12.838)	−0.144	< 0.001
2020	12.742 [0.006] (12.73, 12.755)	−0.227	< 0.001
2021	12.719 [0.007] (12.706, 12.732)	−0.251	< 0.001
2022	12.603 [0.007] (12.590, 12.617)	−0.366	< 0.001
2023	12.584 [0.007] (12.570, 12.597)	−0.386	< 0.001
Overall *P* value for trend^b^	0.005		

2015 served as the reference year for all annual comparisons.

^a^*P* value for difference in mean vitamin levels was calculated using the linear combinations of parameters.

^b^*P* value for overall trend was calculated using the partial Mann-Kendall trend test.

**Figure 1:**
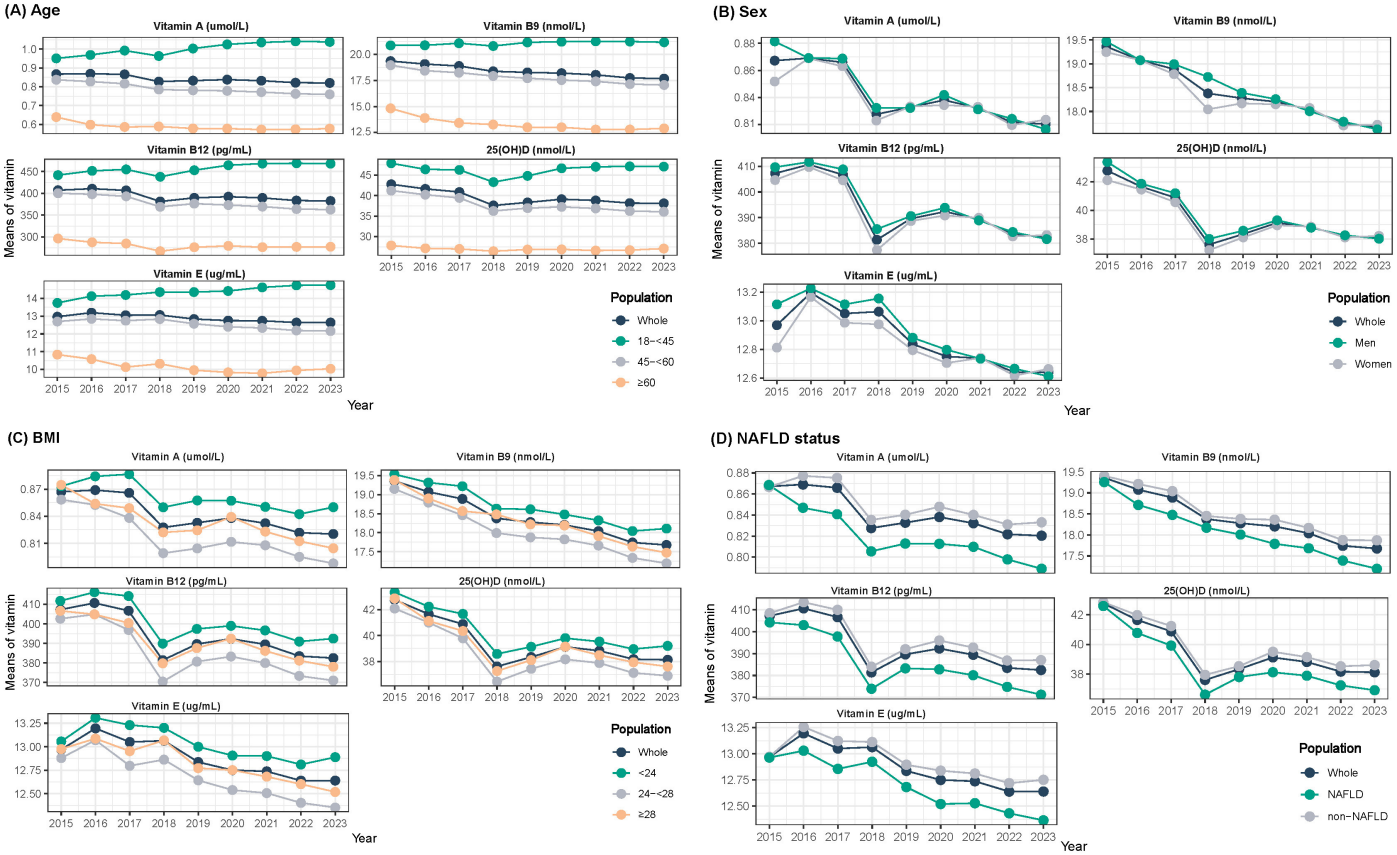
Temporal trends of serum vitamin profiles by **(A)** age, **(B)** sex, **(C)** BMI, and **(D)** non-alcoholic fatty liver disease status, 2015–2023.

Temporal trends in serum vitamin profiles across calendar years were also summarized by stratification by age, sex, BMI, and NAFLD status (Figure 1). By age groups, serum vitamin levels decreased with increasing age. Adults with age < 45 years had the highest vitamin levels, and in sharp contrast, the lowest vitamin levels were consistently seen for participants with age ≥60 years. Generally, vitamin levels were lower in women than in men, with the sex difference gradually narrowing from 2015 to 2016 and becoming comparable thereafter. By BMI group, vitamin levels were highest among participants with BMI < 24 kg/m^2^, followed by those with BMI >28 kg/m^2^ and then those with BMI 24–28 kg/m^2^. By NAFLD status, participants with NAFLD had the lowest vitamin levels, with downward temporal trends.

### 3.3 Associations between vitamin profiles and NAFLD

**Figure 2:**
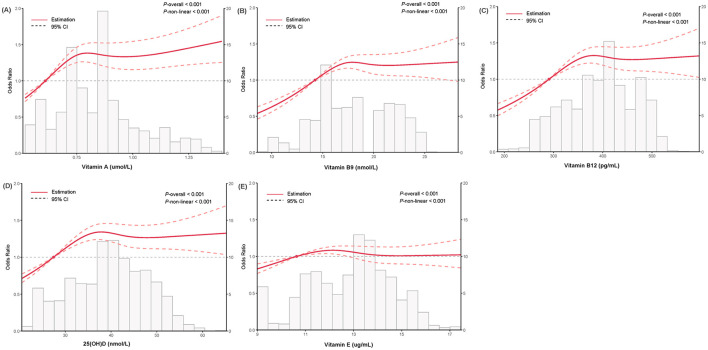
Dose-response relationship between serum vitamin profiles and non-alcoholic fatty liver disease.

Among 67,356 participants, 18,533 (27.515%) had NAFLD. Lower vitamin levels were associated with increased risk of NAFLD (Table 3). For vitamin A, compared with the Q1 group, the multivariable-adjusted ORs and 95% CIs for NAFLD across subsequent quintile groups were 1.194 (1.107, 1.289), 1.194 (1.085, 1.313), 1.133 (1.001, 1.282), and 1.120 (0.983, 1.275), sequentially. Corresponding ORs (95% CIs) for vitamin B9 were 1.156 (1.071, 1.247), 1.165 (1.060, 1.279), 1.021 (0.912, 1.143), and 1.073 (0.947, 1.216). ORs (95% CIs) for vitamin B12 were 1.259 (1.167, 1.360), 1.203 (1.095, 1.322), 1.169 (1.043, 1.310), and 1.150 (1.013, 1.305). For 25(OH)D, ORs (95% CIs) were 1.205 (1.119, 1.298), 1.246 (1.140, 1.363), 1.140 (1.020, 1.274), and 1.135 (1.003, 1.284). ORs (95% CIs) for vitamin E were 1.167 (1.081, 1.260), 1.177 (1.071, 1.292), 1.035 (0.925, 1.159), and 1.064 (0.940, 1.205). Complementarily, the RCS indicated non-linear dose-response relationships between serum vitamins and NAFLD status (All *P*_non − linear_ relation < 0.01; Figure 2), with quintile-based associations largely consistent with the non-significant intervals of the RCS curves.

**Table 3:** Associations of non-alcoholic fatty liver disease according to the quintiles of serum vitamin profiles.

Group	No. (cases)	Model 1		Model 2		Model 3	
		OR (95% CI)	*P* value	OR (95% CI)	*P* value	OR (95% CI)	*P* value
Vitamin A (μmol/L)							
Q1 [0.45, 0.70]	13,706 (4,117)	1 (Reference)		1 (Reference)		1 (Reference)	
Q2 (0.70, 0.77]	13,651 (4,264)	1.440 (1.353, 1.532)	< 0.001	1.405 (1.320, 1.496)	< 0.001	1.194 (1.107, 1.289)	< 0.001
Q3 (0.77, 0.87]	16,590 (4,763)	1.556 (1.444, 1.677)	< 0.001	1.498 (1.389, 1.617)	< 0.001	1.194 (1.085, 1.313)	< 0.001
Q4 (0.87, 0.97]	10,101 (2,422)	1.415 (1.286, 1.557)	< 0.001	1.357 (1.232, 1.495)	< 0.001	1.133 (1.001, 1.282)	0.049
Q5 (0.97, 1.40]	13,308 (2,967)	1.418 (1.284, 1.567)	< 0.001	1.357 (1.228, 1.501)	< 0.001	1.120 (0.983, 1.275)	0.088
Vitamin B9 (nmol/L)							
Q1 [8.60, 15.40]	13,493 (4,079)	1 (Reference)		1 (Reference)		1 (Reference)	
Q2 (15.40, 18.39]	13,484 (4,200)	1.387 (1.304, 1.475)	< 0.001	1.353 (1.272, 1.440)	< 0.001	1.156 (1.071, 1.247)	< 0.001
Q3 (18.39, 19.34]	13,610 (3,915)	1.491 (1.383, 1.607)	< 0.001	1.435 (1.330, 1.548)	< 0.001	1.165 (1.060, 1.279)	0.001
Q4 (19.34, 21.06]	13,305 (3,248)	1.332 (1.219, 1.456)	< 0.001	1.275 (1.165, 1.395)	< 0.001	1.021 (0.912, 1.143)	0.716
Q5 (21.06, 28.26]	13,464 (3,091)	1.369 (1.243, 1.508)	< 0.001	1.307 (1.186, 1.442)	< 0.001	1.073 (0.947, 1.216)	0.270
Vitamin B12 (pg/mL)							
Q1 [174.09, 321.58]	13,473 (4,003)	1 (Reference)		1 (Reference)		1 (Reference)	
Q2 (321.58, 379.85]	13,470 (4,293)	1.472 (1.383, 1.566)	< 0.001	1.435 (1.348, 1.527)	< 0.001	1.259 (1.167, 1.360)	< 0.001
Q3 (379.85, 413.18]	13,472 (3,908)	1.504 (1.396, 1.621)	< 0.001	1.448 (1.342, 1.562)	< 0.001	1.203 (1.095, 1.322)	< 0.001
Q4 (413.18, 465.23]	13,475 (3,382)	1.454 (1.330, 1.589)	< 0.001	1.390 (1.270, 1.521)	< 0.001	1.169 (1.043, 1.310)	0.007
Q5 (465.23, 613.97]	13,466 (2,947)	1.365 (1.238, 1.505)	< 0.001	1.301 (1.179, 1.436)	< 0.001	1.150 (1.013, 1.305)	0.031
25(OH)D (nmol/L)							
Q1 [14.03, 31.22]	13,494 (3,934)	1 (Reference)		1 (Reference)		1 (Reference)	
Q2 (31.22, 38.11]	13,457 (4,321)	1.438 (1.354, 1.528)	< 0.001	1.405 (1.322, 1.494)	< 0.001	1.205 (1.119, 1.298)	< 0.001
Q3 (38.11, 41.96]	13,473 (3,983)	1.553 (1.445, 1.668)	< 0.001	1.499 (1.394, 1.612)	< 0.001	1.246 (1.140, 1.363)	< 0.001
Q4 (41.96, 47.32]	13,469 (3,191)	1.361 (1.247, 1.486)	< 0.001	1.305 (1.194, 1.426)	< 0.001	1.140 (1.020, 1.274)	0.021
Q5 (47.32, 65.18]	13,463 (3,104)	1.388 (1.262, 1.526)	< 0.001	1.328 (1.206, 1.462)	< 0.001	1.135 (1.003, 1.284)	0.044
Vitamin E (μg/mL)							
Q1 [8.77, 11.25]	13,548 (4,084)	1 (Reference)		1 (Reference)		1 (Reference)	
Q2 (11.25, 12.66]	13,440 (4,206)	1.415 (1.332, 1.504)	< 0.001	1.381 (1.299, 1.469)	< 0.001	1.167 (1.081, 1.260)	< 0.001
Q3 (12.66, 13.47]	13,638 (3,933)	1.514 (1.408, 1.628)	< 0.001	1.461 (1.358, 1.572)	< 0.001	1.177 (1.071, 1.292)	< 0.001
Q4 (13.47, 14.36 ]	13,265 (3,196)	1.364 (1.252, 1.485)	< 0.001	1.307 (1.198, 1.425)	< 0.001	1.035 (0.925, 1.159)	0.550
Q5 (14.36, 18.13]	13,465 (3,114)	1.382 (1.260, 1.516)	< 0.001	1.322 (1.204, 1.451)	< 0.001	1.064 (0.940, 1.205)	0.324

Model 1: Adjusted for the year of health examination, age, and sex.

Model 2: Model 1 + race and marital status.

Model 3: Model 2 + BMI group, hypertension, diabetes, and dyslipidemia.

The combined effects of serum vitamin B9 and B12 on NAFLD risk were also evaluated (Supplementary Table S2). In the fully adjusted model, high vitamins B9 and high B12 group showed a significantly reduced NAFLD prevalence compared with the low B9 and low B12 group.

## 4 Discussion

This study investigated 2015–2023 temporal trends in serum vitamins A, B9, B12, 25(OH)D, and E among Chinese adults undergoing health examinations, and explored their associations with NAFLD prevalence. The key findings revealed that serum levels of vitamins A, B9, B12, 25(OH)D, and E declined over the nine-year period, particularly in women, individuals aged ≥60 years, and those with overweight/obesity or NAFLD. Lower vitamin levels were associated with an increased risk of NAFLD, and restricted cubic spline (RCS) analyses demonstrated non-linear dose-response relationships between all five serum vitamins and NAFLD status.

The observed decreases in vitamins A, B9, B12, and E align with global micronutrient status shifts, though regional variations persist (41, 42). Serum vitamin levels were lowest in women, adults aged ≥60 years, and those with overweight/obesity or NAFLD, indicating these subgroups need early attention and priority intervention. These disparities relate to biological and behavioral factors: elderly adults have reduced nutrient absorption and intake (43), the narrower sex gap in vitamins A, D, and E after 2016 may reflect the impacts of released *Chinese Dietary Guidelines (2016)* with scientific and reasonable dietary advice for the general population and sex-specific groups (44), and obesity lowers circulating vitamins via adipose sequestration and altered metabolism (45). These findings identify high-priority groups for targeted interventions like vitamin supplementation or dietary counseling to mitigate deficiency risks. Addressing declines via targeted policies remains vital, highlighting the public health value of vitamin monitoring.

Lower vitamin levels were associated with an increased risk of NAFLD, and non-linear dose-response relationships were evident in RCS analyses (All *P*_non − linear_ < 0.01). From the perspective of linear associations, a prospective study involving 2658 Chinese adults aged 40–75 years from the Guangzhou Nutrition and Health Study revealed that a higher serum vitamin A concentration was correlated with NAFLD progression (46). A cross-sectional study of 201 subjects in Shanghai, China, with a BMI ≥23 kg/m^2^, found a significant inverse relationship between serum 25(OH)D concentration and severe MASLD (47); however, an MR study including nearly 10,000 community-dwelling Chinese adults from the SPECT-China study suggested no causal role of vitamin D in the NAFLD development (32). A possible explanation for those findings contrasts with our study is that most vitamin levels in our health examination population fell within normal reference ranges (serum vitamin A: 0.52–2.2 μmol/L; vitamin B9: 6.8–36.3 nmol/L; vitamin B12: 200–900 pg/mL; 25(OH)D: 25–200 nmol/L; vitamin E: 10–15 μg/mL), limiting variability. Evidence for non-linear associations between serum vitamins and health outcomes primarily derives from the US populations. A prospective cohort study of 6137 US adults from the NHANES database identified a significant U-shaped association between serum vitamin A concentrations and all-cause mortality risk in NAFLD patients, emphasizing the importance of monitoring and maintaining optimal serum vitamin A levels (2). Another retrospective US cohort study demonstrated U-shaped associations of serum vitamin D levels with cirrhosis, hepatocellular carcinoma, and mortality risks (19). In contrast, a cross-sectional analysis of 83,625 participants from Wenzhou, China, revealed an L-shaped relationship between serum vitamin D and metabolic-associated fatty liver disease (48). Given such population-specific discrepancies, potential non-linear relationships between serum vitamin profiles and NAFLD risks warrant further investigation in the Chinese population.

This study has several strengths. First, it systematically examined changes in serum vitamin profiles among a health check-up population over nearly a decade. Capturing longitudinal variations in vitamin levels provided empirical evidence for understanding regional characteristics of serum vitamin status and contributed to knowledge on population-specific micronutrient dynamics. Second, it identified subpopulations with the lowest vitamin levels, stratified by age, sex, obesity status, and NAFLD status. This subgroup analysis aids targeted prevention of vitamin deficiency in high-risk groups and offers clues for early prevention of vitamin-related disease risks, precise health management, and evidence-based clinical decision-making. This study also has limitations. First, regional differences in dietary patterns and lifestyles mean observed serum vitamin trends only reflect Southwest China, limiting generalizability to regions with distinct dietary and lifestyle contexts. Second, the study population's overall serum vitamin levels fell within normal reference ranges, leading to insufficient concentration variability that may have constrained robust confirmation of serum vitamin-NAFLD risk associations. Third, NAFLD was diagnosed qualitatively, preventing exploration or validation of links between serum vitamin levels and NAFLD severity; future studies will use liver elastography (e.g., FibroScan) or magnetic resonance imaging (MRI) to assess NAFLD progression. Fourth, data constraints meant lifestyle-related confounding factors (e.g., smoking, alcohol consumption) were not adjusted for in serum vitamin-NAFLD association analyses, and uncontrolled confounding may bias results, highlighting the need for more comprehensive data collection in future research.

## 5 Conclusions

Our findings reveal a 9-year decreasing trend in serum vitamin A, B9, B12, 25(OH)D, and E levels among Chinese adults in the health examination population, particularly in those aged ≥60 years, and those with overweight/obesity or NAFLD. These results suggest targeted attention should be given to vitamin levels in elderly Chinese women and individuals with metabolic disorders, as this focus may support the development of public health policies and help reduce disease burden.

## Data Availability

The original contributions presented in the study are included in the article/Supplementary material, further inquiries can be directed to the corresponding author.
