# Supplementary material for: Temporal trend of serum vitamin profiles and the association with non-alcoholic fatty liver disease
Source: Front Nutr. 2026 May 8;13:1712392. doi: 10.3389/fnut.2026.1712392 (PMC13193981; doi:10.3389/fnut.2026.1712392)

Supplementary Material

**Table S1. Basic characteristics in the health examination population used in association analysis.**

| **Characteristics** | **Whole sample** | **Non-NAFLD** | **NAFLD** | ***P* value** |
| --- | --- | --- | --- | --- |
|  | **(N=67,356)** | **(N=48,823)** | **(N=18,533)** |  |
| **Age, year** | 47.066 (12.391) | 46.311 (12.788) | 49.054 (11.034) | <0.001 |
| **Age group** |  |  |  |  |
| 18–<44 | 26,839 (39.846%) | 20,871 (42.748%) | 5968 (32.202%) | <0.001 |
| 45–<60 | 30,493 (45.271%) | 20,871 (42.748%) | 9622 (51.918%) |  |
| ≥60 | 10,024 (14.882%) | 7081 (14.503%) | 2943 (15.880%) |  |
| **Men, n (%)** | 32,795 (48.689%) | 19,874 (40.706%) | 12,921 (69.719%) | <0.001 |
| **Chinese Han, n (%)** | 65,863 (97.783%) | 47,748 (97.798%) | 18,115 (97.745%) | 0.673 |
| **Married/Cohabit, n (%)** | 25,993 (38.590%) | 18,662 (38.224%) | 7331 (39.556%) | <0.001 |
| **Vitamin A (umol/L)** | 0.842 (0.183) | 0.850 (0.186) | 0.822 (0.174) | <0.001 |
| **Vitamin B9 (nmol/L)** | 18.425 (3.238) | 18.544 (3.262) | 18.113 (3.155) | <0.001 |
| **Vitamin B12 (pg/mL)** | 394.022 (71.793) | 396.919 (72.417) | 386.388 (69.549) | <0.001 |
| **25(OH)D (nmol/L)** | 39.582 (8.337) | 39.873 (8.398) | 38.815 (8.124) | <0.001 |
| **Vitamin E (ug/mL)** | 12.868 (1.752) | 12.932 (1.759) | 12.699 (1.723) | <0.001 |
| **BMI (kg/m^2^)** | 24.303 (3.500) | 23.192 (2.995) | 27.210 (3.025) | <0.001 |
| **BMI group** |  |  |  |  |
| <24 | 31,509 (48.484%) | 29,354 (62.419%) | 2155 (11.998%) | <0.001 |
| 24–<28 | 24,640 (37.914%) | 15,185 (32.290%) | 9455 (52.639%) |  |
| ≥28 | 8840 (13.602%) | 2488 (5.291%) | 6352 (35.364%) |  |
| **WC (cm)** | 82.729 (10.347) | 79.299 (8.968) | 91.706 (8.088) | <0.001 |
| **SBP (mmHg)** | 124.604 (18.449) | 121.726 (17.949) | 132.178 (17.579) | <0.001 |
| **DBP (mmHg)** | 77.381 (12.378) | 75.273 (11.767) | 82.928 (12.230) | <0.001 |
| **Fasting glucose (mg/dL)** | 103.365 (27.101) | 99.224 (20.915) | 114.237 (36.759) | <0.001 |
| **TC (mg/dL)** | 196.919 (39.013) | 193.131 (37.661) | 206.857 (40.713) | <0.001 |
| **TG (mg/dL)** | 150.849 (126.003) | 121.201 (85.858) | 228.895 (172.805) | <0.001 |
| **LDL-C (mg/dL)** | 112.739 (28.428) | 109.911 (27.933) | 120.109 (28.385) | <0.001 |
| **HDL-C (mg/dL)** | 52.437 (12.352) | 54.831 (12.297) | 46.202 (10.117) | <0.001 |
| **Hypertension, n (%)** | 13,574 (22.304%) | 7602 (17.238%) | 5972 (35.635%) | <0.001 |
| **Diabetes, n (%)** | 4877 (7.337%) | 1739 (3.613%) | 3138 (17.115%) | <0.001 |
| **Dyslipidemia, n (%)** | 23,141 (35.156%) | 11,964 (25.159%) | 11,177 (61.177%) | <0.001 |
| **GGT (IU/L)** | 36.718 (47.378) | 28.612 (35.26) | 57.976 (65.166) | <0.001 |
| **AST (IU/L)** | 25.839 (18.693) | 24.449 (18.887) | 29.484 (17.662) | <0.001 |
| **ALT (IU/L)** | 26.660 (24.846) | 22.610 (23.144) | 37.280 (25.997) | <0.001 |
| **ALP (IU/L)** | 84.505 (27.531) | 82.262 (27.706) | 90.387 (26.171) | <0.001 |

Apart from major independent variables (e.g., vitamin A level), some variables contained missing data.

*P* values indicate group differences in all indicators between non-NAFLD and NAFLD groups, analyzed by *t*-tests (continuous variables) and Chi-square tests (categorical variables).

**Table S2. The combined effects of serum vitamin B9 and B12 on NAFLD prevalence.**

| **Group** | **No.(cases)** | **Model 1** | |  | **Model 2** | |  | **Model 3** | |
| --- | --- | --- | --- | --- | --- | --- | --- | --- | --- |
|  |  | **OR (95% CI)** | ***P* value** |  | **OR (95% CI)** | ***P* value** |  | **OR (95% CI)** | ***P* value** |
| **Serum vitamin B9 & vitamin B12** |  |  |  |  |  |  |  |  |  |
| Low vitamin B9 & low vitamin B12 | 31,633 (9713) | 1 (Reference) | 1 |  | 1 (Reference) | 1 |  | 1 (Reference) | 1 |
| Low vitamin B9 & high vitamin B12 | 1994 (563) | 1.081 (0.971, 1.202) | 0.154 |  | 1.062 (0.954, 1.181) | 0.271 |  | 1.049 (0.919, 1.196) | 0.481 |
| High vitamin B9 & low vitamin B12 | 2035 (605) | 1.057 (0.950, 1.176) | 0.306 |  | 1.038 (0.932, 1.155) | 0.491 |  | 0.862 (0.749, 0.991) | 0.037 |
| High vitamin B9 & high vitamin B12 | 31,694 (7652) | 0.961 (0.908, 1.018) | 0.174 |  | 0.940 (0.887, 0.995) | 0.032 |  | 0.912 (0.849, 0.980) | 0.012 |

Notes: Vitamin levels were dichotomized into low and high groups based on median values. Low vitamin B9: ≤ 19.04 nmol/L; Low vitamin B12: ≤ 401.5 pg/mL.

Model 1: Adjusted for the year of health examination, age, and sex.

Model 2: Model 1 + race and marital status.

Model 3: Model 2 + BMI group, hypertension, diabetes, and dyslipidemia.

**Figure S1. Joinpoint regression analysis of temporal trends in serum vitamin A stratified by (A) age, (B) sex, (C) BMI and (D) NAFLD status, 2015–2023**


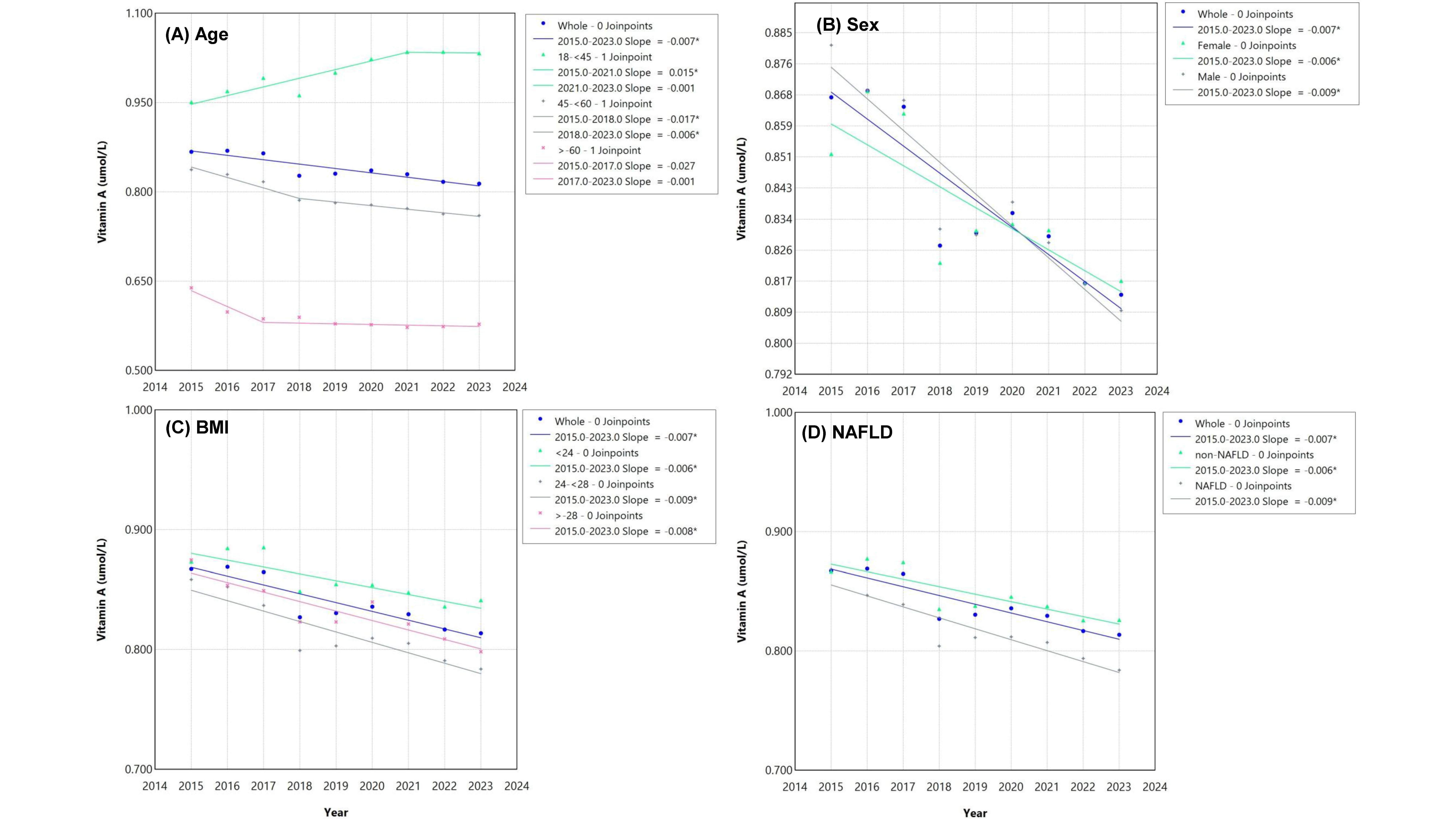


**Figure S2. Joinpoint regression analysis of temporal trends in serum vitamin B9 stratified by (A) age, (B) sex, (C) BMI and (D) NAFLD status, 2015–2023**


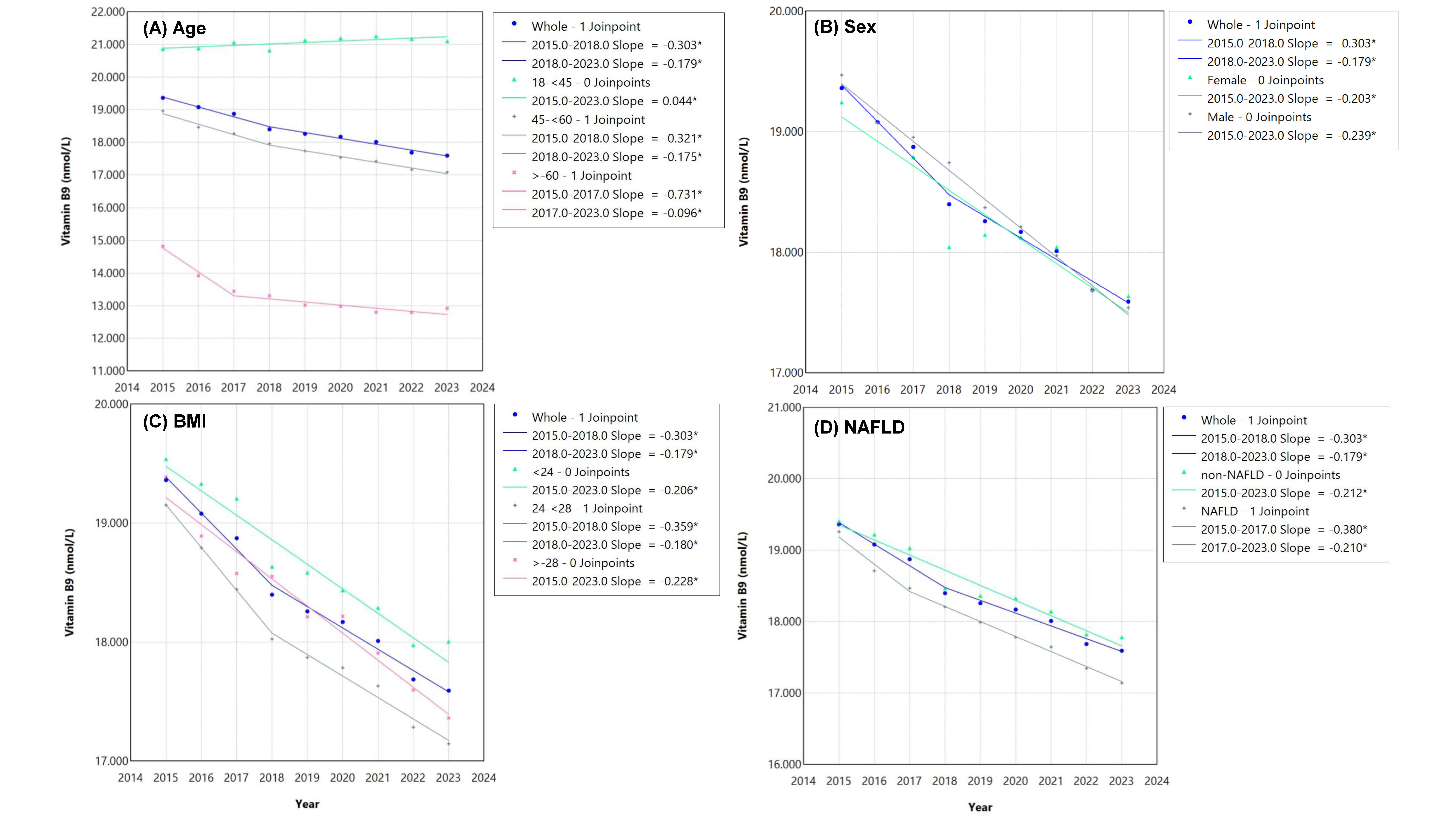


**Figure S3. Joinpoint regression analysis of temporal trends in serum vitamin B12 stratified by (A) age, (B) sex, (C) BMI and (D) NAFLD status, 2015–2023**


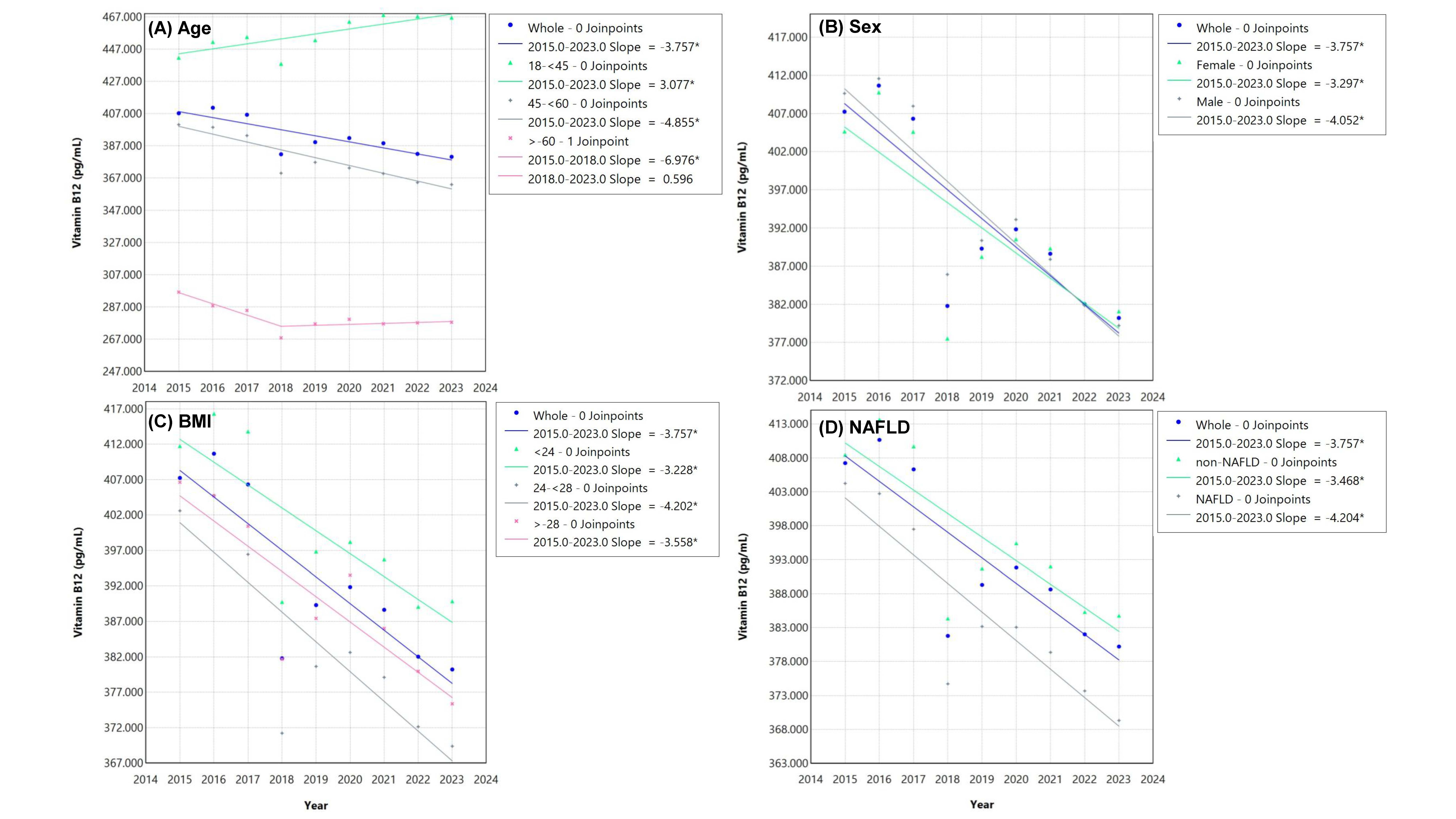


**Figure S4. Joinpoint regression analysis of temporal trends in serum vitamin 25(OH)D stratified by (A) age, (B) sex, (C) BMI and (D) NAFLD status, 2015–2023**


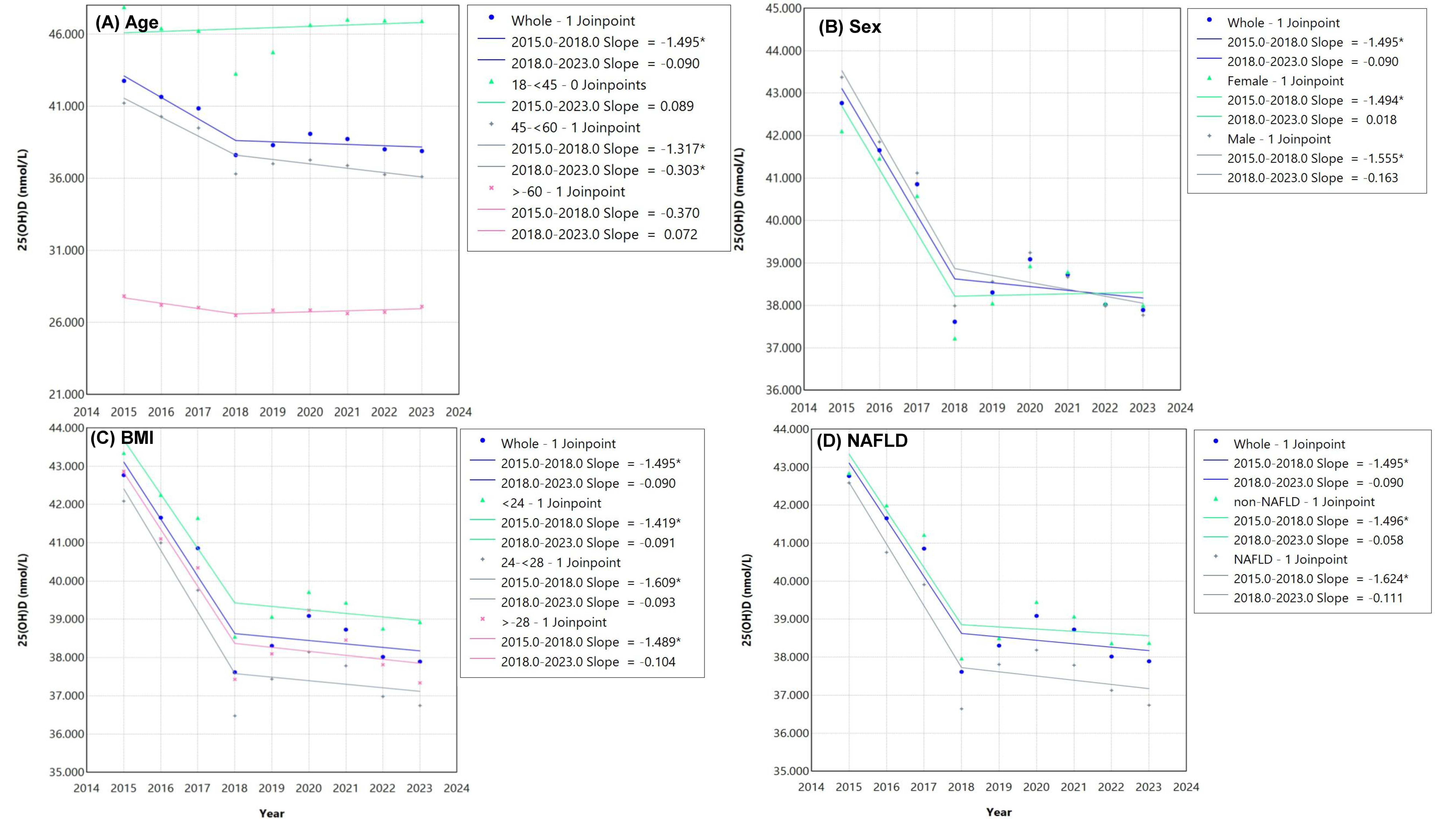


**Figure S5. Joinpoint regression analysis of temporal trends in serum vitamin E stratified by (A) age, (B) sex, (C) BMI and (D) NAFLD status, 2015–2023**


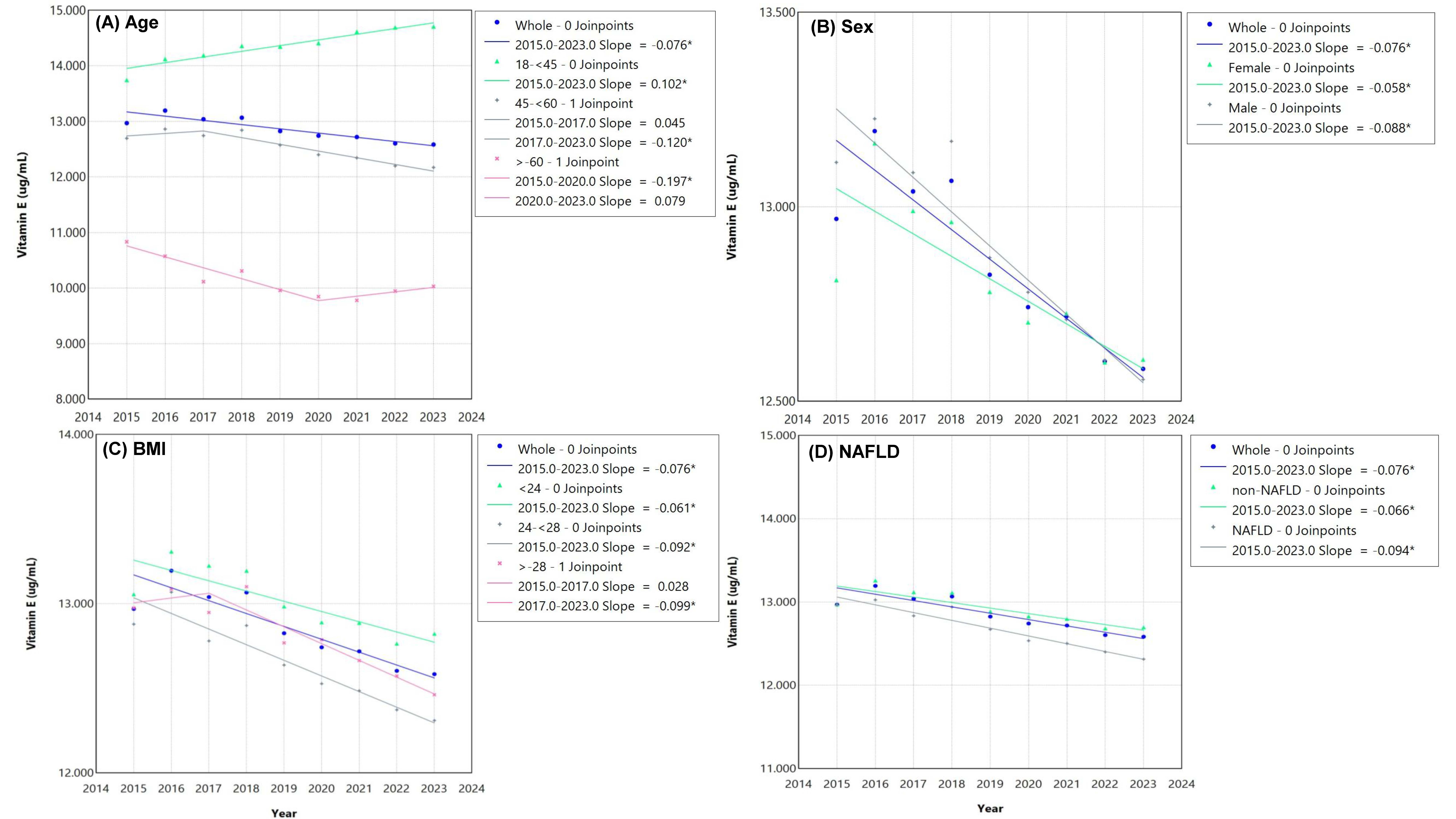

Supplement: Supplementary file 1 [file Table_1.docx]
